# Validation of preoperative predictor score for difficult laparascopic cholecystectomy and a modified intraoperative grading score of the difficulty of laparascopic cholecystectomy: from a resource limited setting

**DOI:** 10.1186/s12893-025-02784-1

**Published:** 2025-01-27

**Authors:** Nurhussen Mossa Ahmed, Surafel Mulatu Djote, Getachew Desta Alemayehu, Wondwossen Amtataw, Sitotaw Mossa Ahmed

**Affiliations:** 1 Department of surgery, Yekatit 12 hospital medical college, Addis Ababa, Ethiopia; 2 Department of statistics, Jinka University, Jinka, Ethiopia

**Keywords:** Preoperative score, Intraoperative score, Difficult laparoscopic cholecystectomy, Symptomatic cholelithiasis

## Abstract

**Background:**

Difficult laparascopic cholecystectomy has greater risk of biliary, vascular and visceral injuries. A tool to predict the difficulty help to prepare a head and avoid complications.

**Aim:**

the aim of this study is validation of preoperative predictor score and a modified intraoperative grading score for difficulty of laparascopic cholecystectomy.

**Methods:**

This study was a cross sectional, hospital based study on 200 patients. There are total of 10 scores for preoperative predictor score and 16 scores for the modified intraoperative grading of LC. Structured checklist questionnaire was used.

**Result:**

prevalence of difficult LC was 40%. age greater than or equal to 50years, history of admission for acute cholecystitis, BMI > 30, palpable GB, impacted stone on imaging, adhesion burying GB, time to identify cystic artery/duct, bile/stone spillage and type of ligature were statistically significantly factors for difficult laparascopic cholecystectomy.

**Conclusion:**

The preoperative scoring is statistically and clinically a good test for predicting the difficult level of laparascopic cholecystectomy (area under ROC = 0.948). The modified intraoperative measure of LC score is a statistically and clinically a good test for classifying the operative outcome of LC (area under ROC = 0.94).

**Supplementary Information:**

The online version contains supplementary material available at 10.1186/s12893-025-02784-1.

## Introduction

Gallstones are an extremely common condition, since they have been found in the gallbladders of Egyptian mummies dating back to 1000 BC [1, 2]. Generally it occurs in approximately 10–20% of the adult population [3]. In USA it has 15% rates [4], 9–21% in Europe [5] and 10% in Japan [6]. More than 80% of gallstones do not cause symptoms, and only 10% and 20% will eventually become symptomatic within 5 years and 20 years of diagnosis [7, 8].

Gallstones are public health problems in Ethiopia. The overall prevalence of gall stone diseases among Hospital admitted patients in referral Hospital of Ethiopia was 10.2% [9] and it accounts for 25.9% of all Gastro Intestinal Unit admissions in Tikur Anbessa Hospital [10].

The two commonly performed types of cholecystectomies are open cholecystectomy and laparoscopic cholecystectomy [11]. Laparoscopic cholecystectomy (LC) since its first description in 1985 is now considered the gold standard for treatment of gall stone disease [12, 13]. LC has clear advantages over the traditional open approach with less postoperative pain, a lower incidence of incisional hernias, less adhesions, smaller scars/less tissue damage, a shorter hospital stay, an earlier return to full activity, a decrease in the overall cost, decreased morbidity, less pain and a quicker recovery [12, 14].

In countries where minimally invasive surgery is advanced, current selection criteria of patients for LC have become more liberal and the absolute contraindications for its performance are patients with uncontrolled coagulopathy, Severe chronic obstructive pulmonary disease, Congestive cardiac failure (ejection fraction < 20%) and patients who have high risk for general anesthesia [11].

Difficult laparoscopic cholecystectomy (DLC) is stressful condition for surgeon which is accompanied by greater risk for various injuries like biliary, vascular and visceral injuries [15].

Multiple factors that may influence the difficulty of a laparascopic cholecystectomy have been described such as age, sex, body mass index (BMI), palpable gall bladder (GB), impacted stone, anatomical variations and previous abdominal surgeries [13, 16–19].

Scoring a value for these factors and developing a tool that predict the difficulty of cholecystectomy can help to choose the best schedule (open or laparoscopic), select the patient according to the level of physician training or to get expert support, inform the patient of the possible difficulty and increase of complications [19].

A number of preoperative scoring systems are reported for acute cholecystitis in well developed countries [15–27], however information regarding a separate preoperative predicting scores for only symptomatic chlelithiasis that can be applied in resource limited setups are scarce. Newly established laparascopic setups and less experienced surgeons usually start laparascopic cholecystectomy on less complicated cases like on symptomatic cholecystectomy and they need separate predictor score of difficulty for such diseases. Our preoperative predictive score for DLC for symptomatic cholelithiasis can fill this gap.

There are two mostly described intraoperative scoring tools to objectively measure the difficulty of laparoscopic cholecystectomy.

The first one was Gupta N et al. and Khetan et al. classification incorporating time taken to finish the laparascopic surgery, Bile/stone spillage, Injury to duct or artery and Conversion to open cholecystectomy [17, 18]. Different limitation of this score are noticed. Some of the variables were subjective like time taken to finish the operation may vary on surgical skills and level of experience. Moreover important operative findings that can strongly affect difficulty of operation like GB adhesion, GB distension/contraction, BMI and previous surgical scar were not included.

The other operative finding score was by Sugrue et al. which incorporates GB adhesion, GB distension, BMI, previous surgery scar, puss/bile outside GB and time taken to identify cystic duct and artery [28]. Surgue et al. score was not an original article instead it is an intraoperative score created from researches done with a purpose to produce a preoperative predictive score of DLC. Moreover important intraoperative findings that can objectively measure DLC like injury to duct/artery and bile/stone spillage were not included in the score.

Our paper creates a modified scoring system to measure the difficulty of LC incorporating comprehensive intraoperative findings such as GB adhesion, presence of GB distension, BMI, adhesion from previous surgery, time taken to identify cystic duct and artery, bile/stone spillage, injury to duct/artery, conversion to open and type of ligature at laparoscopic cholecystectomy. We tried to fill the gaps of both Gupta et al/Khetan et al. and Sugrue et al. scores.

The aim of this study is to define Preoperative predictor score for difficult laparascopic cholecystectomy and to establish a modified intraoperative grading score of the difficulty of laparascopic cholecystectomy.

## Methods

### Study area and period

The study was conducted at Yekatit 12 hospital Medical College and St Paul’s Millennium Medical College, Addis Ababa, Ethiopia. Yekatit 12 Hospital Medical College serves the community for more than 100 years with current catchment population of more than five million. The college starts laparoscopic cholecystectomy for symptomatic cholelithiasis two years back. LC was being done by one laparoscopic trained General surgeon and one hepatobiliary Surgeon. St Paul’s Millennium Medical College has inpatient capacity of more than 700 beds treating an average of 1200 emergency and outpatient clients daily and two trained laparoscopic hepatobiliary surgeons involved in the LC during the study period. Mostly clips were used but when laparoscopic clips were not available extracorporeal suture ligation of the cystic duct and artery was done. The study period was from August 1, 2022 to July 30/2024.

### Study design

This study is a prospective cross sectional, hospital based study. Because patients are contacted at a point in time when a patient is scheduled for LC we collected preoperative factors and then when operated we took intraoperative findings. There was no long term follow up of cases.

### Study population

All patients with diagnosis of symptomatic cholelithiasis who had had laparoscopic cholecystectomy at Yekatit 12 Hospital Medical College and St Paul’s Millennium Medical College between August 1, 2022 to July 30/2024.

### Inclusion criteria

All patients with symptomatic cholelithiasis including previous treated acute cholecystitis and gallstone pancreatitis who had had elective laparoscopic cholecystectomy at Yekatit 12 Hospital Medical College and St Paul’s Millennium Medical College between august 1, 2022 to July 30/2024.

### Exclusion criteria

Patients with Acute cholecystitis, Gall bladder cancer.

### Data collection procedures

The research team systematically collected data using a modified Check list questionnaires from previous studies [15, 18, 19]. Data was collected by surgical residents. Both preoperative and intraoperative parameters like diagnosis, age, gender, BMI, palpable gall bladder, abdominal scar, impacted stone, Gall bladder appearance, distension/contraction, Adhesions from previous surgery, Time to identify cystic artery and duct, Time taken (minutes) to complete LC, Bile / stone spillage, injury to duct or artery Conversion to open were collected were filled.

### Data analysis procedures

Data was entered in and analyzed using the Statistical Package for the Social Sciences (SPSS) version 26. Percentages and count were utilized for categorical variables. All variables with a *p* < 0.05 in the 95% confidence interval in bivariate analysis are entered to multivariate logistic regression model and analyzed to control for potential confounders. Results were analyzed and presented via a combination of textual, tabular and graphic formats.

### Operational definition

Difficult laparoscopic cholecystectomy (DLC) was characterized by numerous operative difficulties (parameters) incorporating the appearance of the GB, presence of GB distension, BMI, adhesion from previous surgery, and time taken to identify cystic duct and artery. A score of < = 2 would imply mild difficulty, 3–4 moderate, 5–7 severe and 8–10 extreme (Table 1).

**Table 1:** Intraoperative measure of DLC (as a standard Michael Sugrue criteria was taken [28])

Risk factors	Score
Gallbladder appearance (adhesion)	Adhesions < 50% of GB : 1 Adhesions burying GB: 3 No adhesion: 0
Distension/Contraction	Distended GB (or contracted shrivelled GB): 1 Unable to grasp with atraumatic laparoscopic forceps: 1 Stone ≥ 1 cm impacted in Hartman’s Pouch : 1 No distension/contraction of GB = 0
BMI > 30	<=30:0 > 30:1
Adhesions from previous surgery limiting access	No:0 Yes:1
Bile or Pus outside GB	No:0 Yes:1
Time	Time to identify cystic artery and duct > 90 min : 1
Total maximum	10

Preoperative predictors score for DLC incorporates age, gender, history of admission for acute cholecystitis, body mass index (BMI), palpable gall bladder (GB), abdominal scar and impacted stone. Score 0–2 is no risk, 3–7 is moderate risk and 8–11 is high risk (Table 2).

**Table 2:** Preoperative risks score for DLC (our modification from Randhawa and Pujahari and Hassan [22, 25] scoring system to fit for symptomatic cholelithiasis

Risk factors	Minimum	Maximum	Total score
Age	< =50 (0)	> 50 (1)	1
Sex	Female (0)	Male (1)	1
History of hospitalization for acute cholecystitis	No)0)	Yes(4)	4
Clinical			
BMI	<=30 (0)	> 30(1)	1
Palpable GB	No (0)	Yes (1)	1
Abdominal scar	No (0)	Infraumblical (1) Supraumblical (2)	2
Sonography			
Impacted stones	No (0)	Yes (1)	1

Intraoperative factors of difficult LC incorporates the appearance of the GB, presence of GB distension, BMI, adhesion from previous surgery, time taken to identify cystic duct and artery, bile/stone spillage, injury to duct/artery, conversion to open and type of ligature. A score of 0–3 would imply mild difficulty, 4–7 moderate, 8–11 severe and 12–16 extreme (Table 3).

**Table 3:** Intraoperative measure of DLC(our modified score from [18, 25, 28]

Risk factors	Score
Gallbladder appearance (adhesion)	Adhesions < 50% of GB : 1 Adhesions burying GB: 3 No adhesion: 0
Distension/Contraction	Distended GB (or contracted shriveled GB): 1 Unable to grasp with atraumatic laparoscopic forceps: 1 Stone ≥ 1 cm impacted in Hartman’s Pouch : 1 No distension/contraction of GB = 0
BMI > 30	<=30:0 > 30:1
Adhesions from previous surgery limiting access	No: 0 Yes:1
Time	Time to identify cystic artery and duct > 90 min : 1
Bile / stone spillage	No: 0 Yes:1
injury to duct or artery	No: 0 Duct only:1 Both:2
Conversion to open	No: 0 Yes:3
Ligature	Clip = 0 Stitch = 1
Total maximum	16

## Result

Of the 200 patients included in this study 185 (92.5%) patients were female and 15(7.5%) were males. The mean age of participants was 47.3 ± 11. The majority of patients were in the age group of < 50 years (*N* = 126, 63%). From the calculated BMI of patients, 76.5%( 153) were having BMI of less than or equal to 30. Those who had history of hospital admission for acute cholecystitis account 21%( *N* = 42). History of previous surgery was noted in 19 patients. It included infraumblical of 8% (16 patients) and supraumblical 1.5%( 3 patients). Impacted stone on imaging was noted in 30(15%) patients. Bile/stone spillage was identified in 37 (18.5%) cases which were promptly managed with saline irrigation and suction and stones picked with laparoscopic forceps (Table 4).

**Table 4:** Distribution of parameters

Parameters	Characteristics	Count	Percent (%)
GB appearance/adhesion	No adhesion	124	62.0
	adhesion < 50% of GB	65	32.5
	Adhesion burying GB	11	5.5
Distended/contracted GB	no distension or contraction of GB	143	71.5
	Distended GB (or contracted shriveled GB)	42	21.0
	stone > 1 cm impacted in Hartmans pouch	9	4.5
	unable to grasp with atraumatic laparascopic forceps	6	3.0
Time to identify cystic artery/duct	<=90	170	85.0
	> 90	30	15.0
Injury to Duct/artery	No	199	99.5
	Yes(duct only)	1	0.5
Ligature	Clip	127	63.5
	Stitch	73	36.5
DLC score	Mild difficulty(0–2)	141	70.5
	Moderate(3–4)	39	19.5
	Severe difficulty(5–70	17	8.5
	Extreme(8–10)	3	1.5

**Fig. 1:**
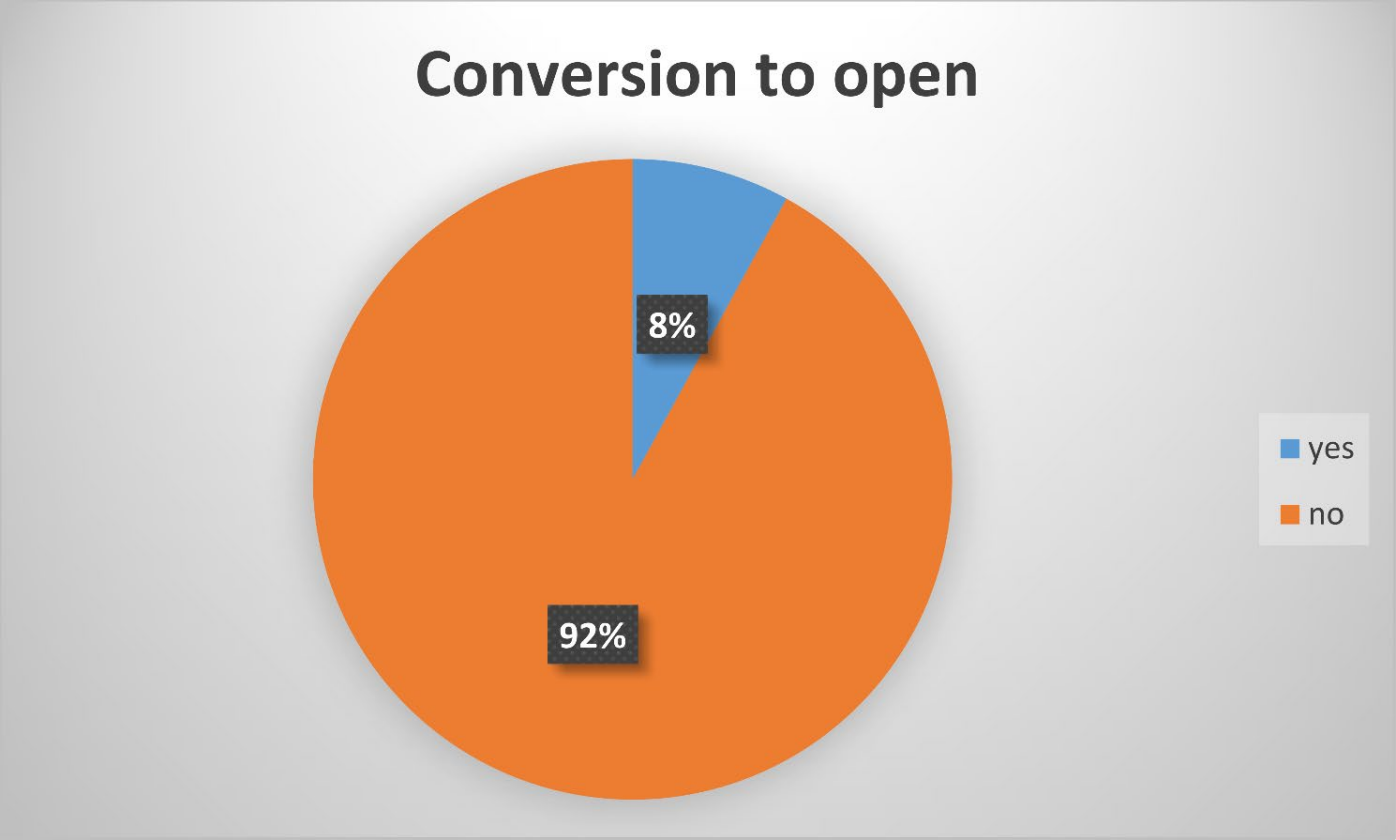
Shows prevalence of conversion to open

There were total 16(8%) cases converted to open in our study all because of dense adhesions at calot’s triangle (Fig. 1).

**Fig. 2:**
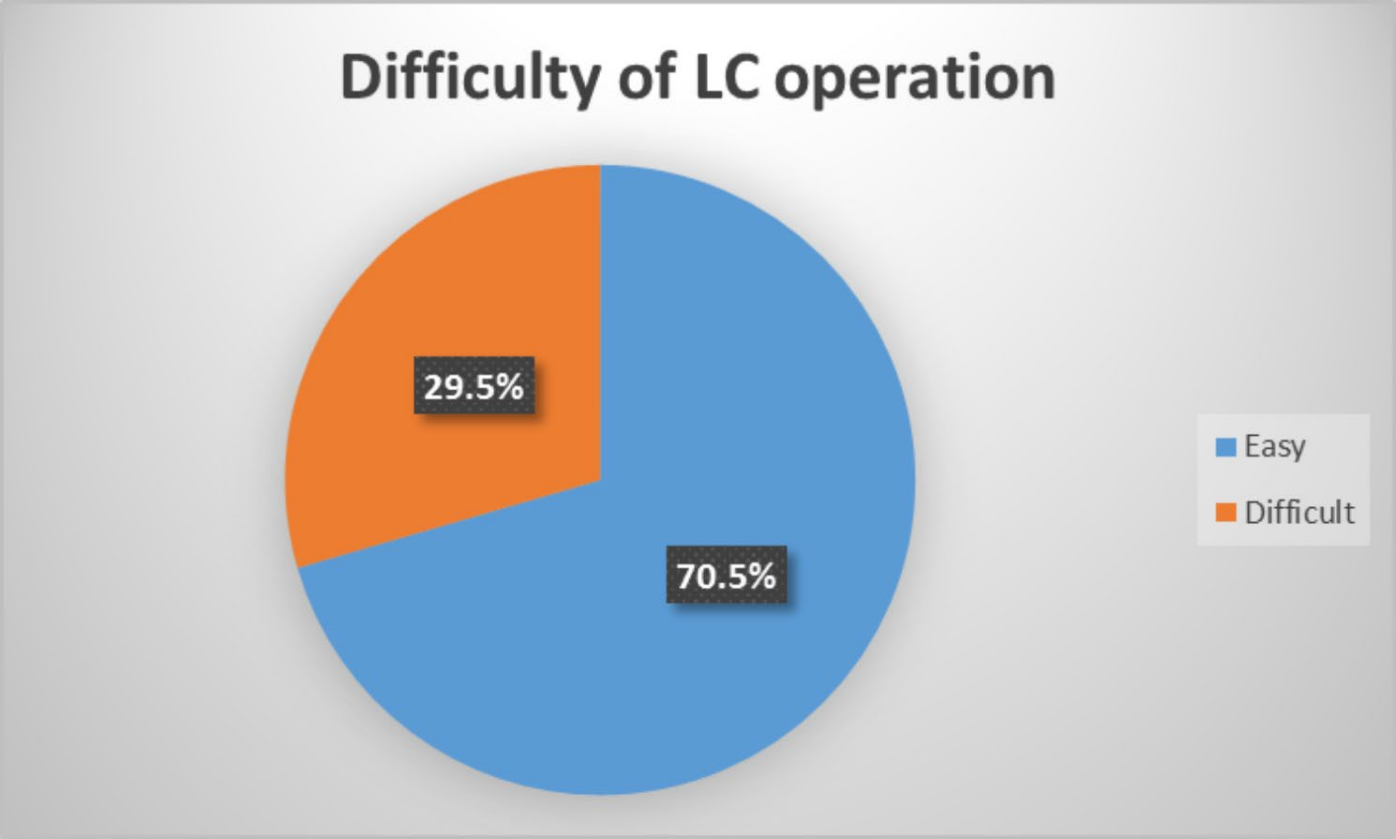
Shows prevalence of difficult laparascopic cholecystectomy

The LC operation outcome showed 70.5% (141) were easy and 29.5% (59) difficult (Fig. 2).

In the preoperative score 69%( 138), 29%( 58) and 2%( 4) were scored easy, difficult and very difficult groups respectively. For the purpose of analysis and interpretation we reorganize the preoperative score into easy and difficult. The relation between the prediction of the difficulty level of the cases preoperatively and the actual outcome of the cases is shown in (Table 5).

**Table 5:** Preoperative evaluation score index in 200 patients with LC

Preoperative Evaluation	LC Operation		Total
	Difficult	Not difficult	
Difficult	64	4	68
Not difficult	3	129	132
Total	67	133	200

Statistical measures of the performance of Our preoperative Score

Sensitivity : 0.955

Specificity :0.969

Positive predictive value(PPV) : 0.941

Negative predictive value(NPV) :0.977

**Fig. 3:**
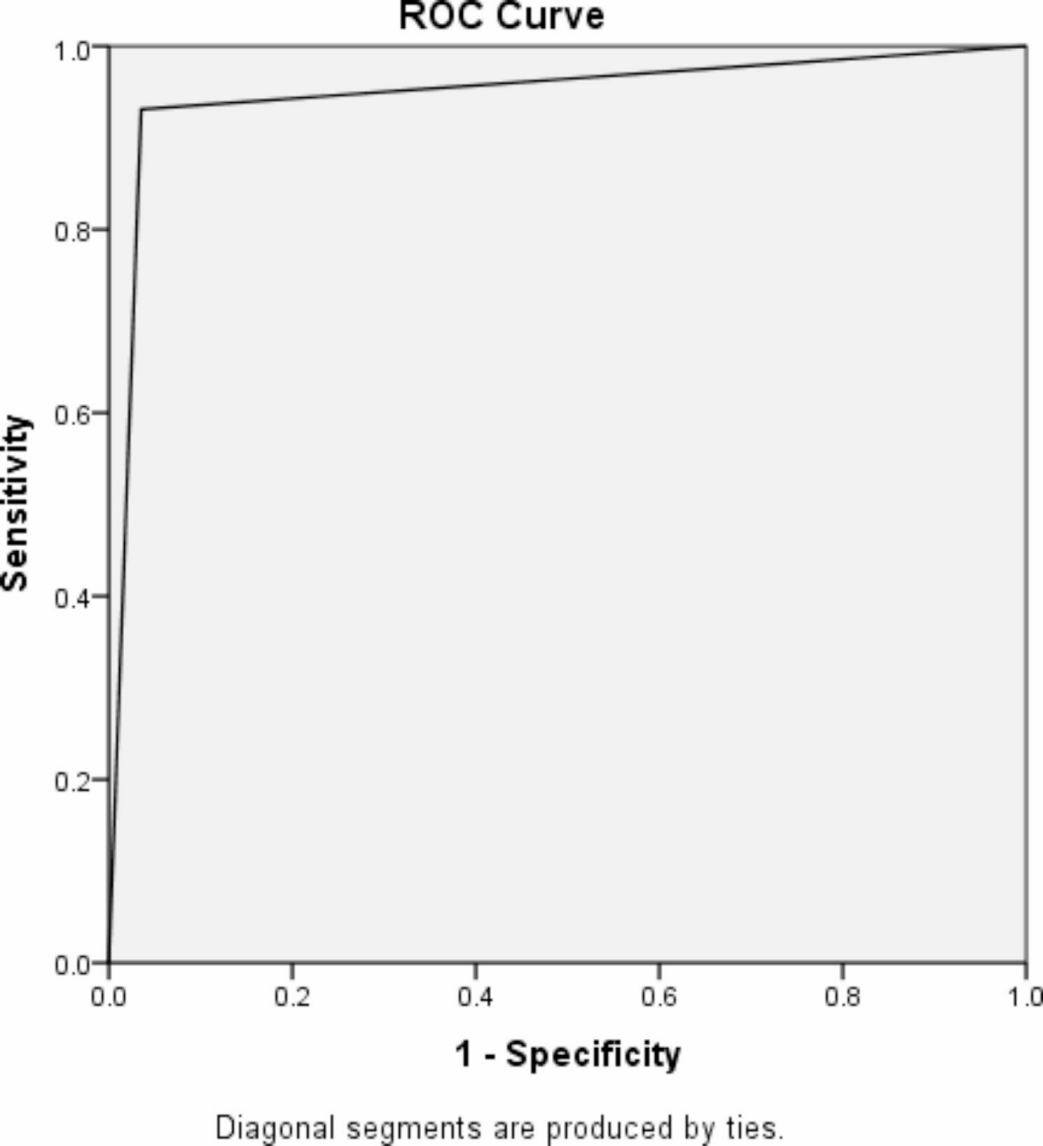
ROC curve and its area under curve for predicting the operative outcome based on preoperative scores

Area under receiving operating characteristics (ROC) curve = 0.948 (Fig. 3).

In the intraoperative score 70.5%( 141), 24.5%( 49) and 5.0%( 10) were scored easy, moderate and severe difficulty respectively. For the purpose of analysis and interpretation we then reorganize the intraoperative score into easy and difficult. The relation between the prediction of the difficulty level of the cases intraoperatively and the actual outcome of the cases is shown in (Table 6).

**Table 6:** Intraoperative evaluation score index in 200 patients with LC

intraoperative Evaluation	LC Operation		Total
	Difficult	Not difficult	
Difficult	57	2	59
Not difficult	2	139	141
Total	59	141	200

Statistical measures of the performance of Our preoperative Score

Sensitivity : 0.966

Specificity :0.985

Positive predictive value(PPV) : 0.966

Negative predictive value(NPV) :0.985

**Fig. 4:**
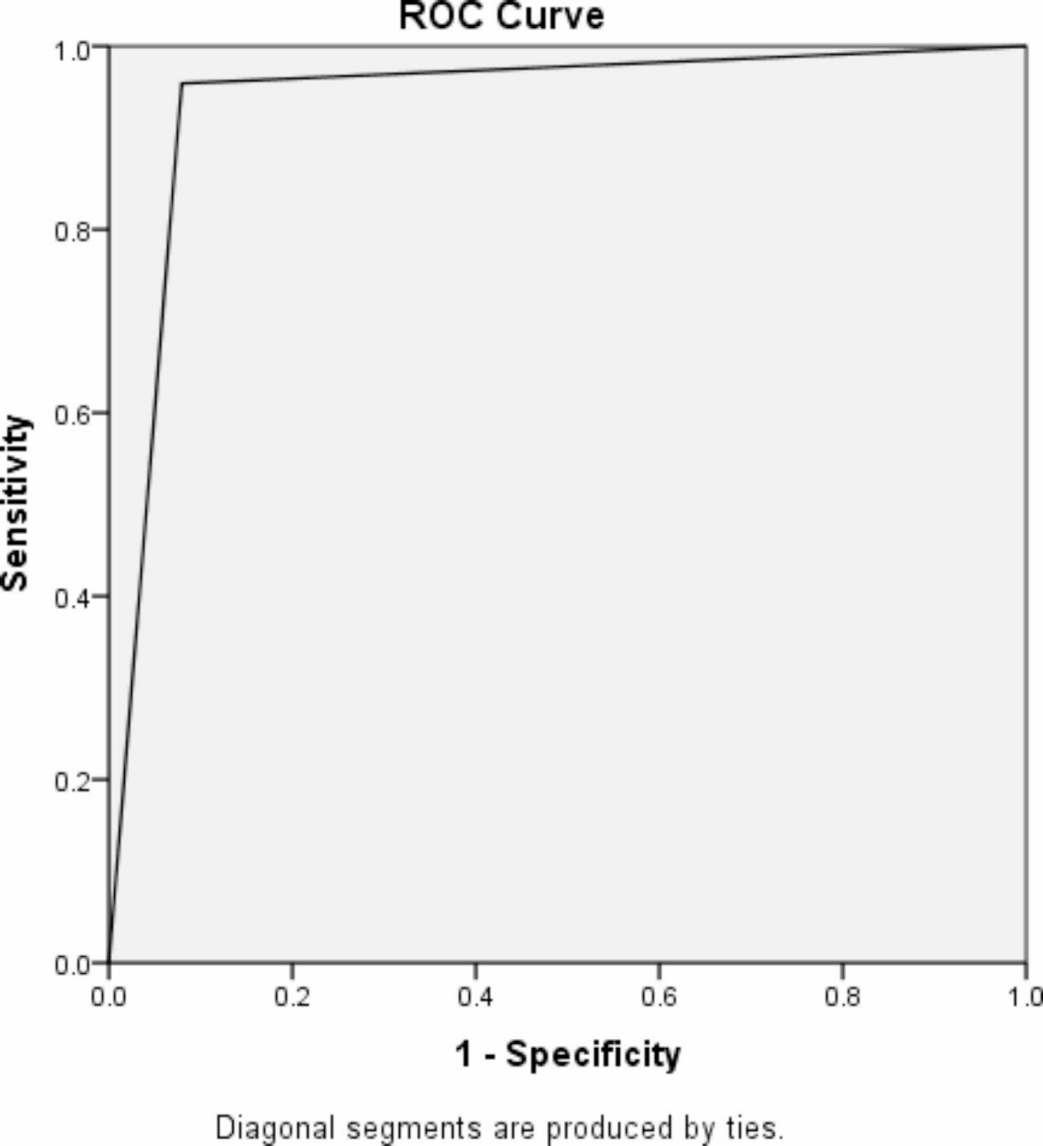
ROC curve and its area under curve for predicting the operative outcome based on intraoperative risk scores

Area under receiving operating characteristics (ROC) curve = 0.94 (Fig. 4).

Operative outcome was correlated with the various preoperative and intraoperative factors included in the scoring system, and data analyzed first by bivariate logistic regression and those with statistical significant on bivariate analysis are fed to multivariate logistic regression to identify factors with statistical significant with outcome variable (Table 7). From our data, we observed that age > 50year, Male sex, history of admission for acute cholecystitis, BMI > 30, palpable GB, impacted stone on imaging, previous abdominal surgical scar, GB appearance/adhesion, time to identify cystic artery/duct, bile/stone spillage, conversion to open cholecystectomy and type of ligature were significantly associated factors in the Bivariable analysis. However on the multivariate logistic regression analysis the risk factors for causing difficulties in laparoscopic cholecystectomy are age > = 50year(*p* < 0.035), history of admission for acute cholecystitis(*p* < 0.0001), BMI > 30(*p* < 0.025), palpable GB(*p* < 0.0001), impacted stone on imaging(*p* < 0.002), adhesion burying GB(*p* < 0.001), time to identify cystic artery/duct(*p* < 0.010), bile/stone spillage(*p* < 0.041) and type of ligature(*p* < 0.005).

**Table 7:** Showing binary logistic regression analysis of factors for difficult laparascopic cholecystectomy

Variable		Multivariable logistic regresion			
		Sig.(*P* value)	AOR	95% C.I.for EXP(B)	
				Lower	Upper
Age	< 50year	1			
	>=50year	**0.035**	**8.7**	**1.1**	**65**
History of hospitalization for acute cholecystitis	No				
	Yes	**0.0001**	**736.**	**23.**	**23,184**
BMI	<=30	**1**			
	> 30	**0.025**	**7.8**	**1.3**	**47.**
Palpable GB	No	1			
	Yes	**0.0001**	**133.**	**8.6**	**2049**
Impacted stone on imaging	No	1			
	Yes	**0.002**	**84.1**	**4.982**	**1421.**
GB appearance/adhesion	No adhesion	1			
	adhesion < 50% of GB	0.001	0.002	0.032	1.09
	Adhesion burying GB	**0.001**	**13.**	**2.9**	**63**
Time to identify cystic artery/duct	<=90	1			
	> 90	**0.010**	**15.**	**1.8**	**123.**
Bile/stone Spillage	No				
	Yes	**0.041**	**4.3**	**1.1**	**18.**
Ligature	Clip	0.001	3	0.563	0.12
	Stitch	**0.005**	**8.6**	**1.9**	**38.**

## Discussion

Age is a risk factor for difficult GB surgery [29]. In the present series, age greater than or equal to 50 years was 8 times more at risk of having difficult laparascopic cholecystectomy than those less than 50years.

Male sex has been described to be associated with difficult LC [26, 30]. In our study, sex was not statistically associated with a high risk of difficult cholecystectomy.

Obesity poses a great challenge to the safe and timely completion of the procedure due to various factors in form of difficulty umbilical port (peritoneal) access, dissection of fatty calot [26, 30]. In our study, we found strong correlation between BMI > 30and difficult level of laparascopic cholecystectomy (*p* < 0.025).

History of acute cholecystitis attacks increases scarring and fibrosis of GB as well as the adhesions at the Calot’s triangle [20]. There is a linear correlation between previous history of hospitalization due to acute attacks of cholecystitis and the difficulty level of LC [31]. These findings are similar to our study where history of an acute attack requiring hospitalisation was one of the main factors for difficulty in laparascopic cholecystectomy(*p* < 0.0001).

Clinically palpable gall bladder could be due to a distended GB, mucocele GB or due to the adhesions between the GB and the omentum [20]. Palpable GB was found to be predictor of difficult LC [25]. Similarly in our study palpable GB was a statistically significant predictor of difficult laparoscopic cholecystectomy (*p* < 0.0001).

While performing LC, stone impacted at the neck of GB poses difficulty to grasp the GB neck to allow adequate retraction to perform dissection at the Calot’s triangle. It is a risk factor for DLC [20, 23]. It was found to be a statistically significant factor in predicting the difficulty of the procedure in our study (*p* < 0.002).

Previous upper abdominal surgery may cause the formation of intraperitoneal adhesions and it was found to be statistically significant factor for difficulty of LC in several studies [17, 18, 20]. In our study 16 patients had history of infraumblical surgery and 3 cases supraumblical scar. All of the 3 supraumblical previous surgical scar had difficult LC but were statistically insignificant.

Patients with adhesions burying gall bladder had high chance of being a DLC [21, 27]. In our study all of 11 patients with adhesion burying the GB had conversion to open and showed a statistical significant association to DLC(*p* < 0.001).

Time needed to identify cystic artery/duct > 90 min were statistically significant association with difficulty of LC with *p* value < 0.010.

Intra-op bile/stone spilage showed significant association with the difficulty of LC operation with *p* value < 0.041.

In laparoscopic cholecystectomy, ligation of cystic duct and cystic artery with clips takes less time than by silk suture. Application of stitch takes statistically significant time than clip [32]. In our study stitch had a statisticaly significant association with difficulty of LC operation(*P* < 0 0.005).

In Our study, the preoperative scoring system has a Sensitivity of 95.5%, specificity of 96.9%, PPV of 94.1% and NPV of 97.7% and AUC of 0.948, which showed a score with high sensitivity, specificity and excellent area under ROC curve(> 0.9).

Interpretation of Area under the curve (AUC): 0.9 ≤ AUC: Excellent, 0.8 ≤ AUC < 0.9: Good, 0.7 ≤ AUC < 0.8: Fair, 0.6 ≤ AUC < 0.7: Poor, 0.5 ≤ AUC < 0.6: Fail. For a diagnostic test to be meaningful, the AUC must be greater than 0.5. Generally, an AUC ≥ 0.8 is considered acceptable [33]. Based on this our study AUC is o.959 which is excellent.

Our preoperative score validity tests are comparable to study in Delhi, With sensitivity, specificity PPV and AUC of 95.74%, 73.68%, 88% 0.86 respectively (Gupta 2013), and to a study in Columbia where area under ROC curve was 0.88. The ideal cutoff was 8, with a sensitivity of 75.15%,, specificity of 88.31%,, PPV of 87.32, NPV of 76.83%, and AUC of 88 (Camilo R 2022).

Our modified intraoperative measure of the difficulty of laparascopic cholecystectomy scoring system compared to Surgrue [28] has a Sensitivity of 96.6%, specificity of 98.5%, PPV of 96.6% and NPV of 98.5% and AUC of 0.94. which showed a score with high sensitivity, specificity and excellent area under ROC curve(> 0.9).

## Conclusion

Older age, history of admission for acute cholecystitis, Higher BMI, palpable GB and impacted stone on imaging, GB adhesion, time to identify cystic artery/duct, bile/stone spillage and type of ligature were found statistically significant factors for difficult LC.

The preoperative scoring is statistically and clinically a good test for predicting the difficult level of laparascopic cholecystectomy (area under ROC = 0.948).

The modified intraoperative measure of LC score is a statistically and clinically a good test for classifying the operative outcome of LC (area under ROC = 0.94).

### Limitation of the study

Among the limitations of the study are the subjectivity of some of intraoperative findings such as Gallbladder adhesion and conversion to open. We tried to reduce it by excluding cholecystectomies done by general surgeons who are not trained laparascopic surgery. The sample size is smaller. Large sample size study is required especially for our modified intraoperative score which is less investigated even in previous studies.

## Electronic supplementary material

Below is the link to the electronic supplementary material.

Supplementary Material 1

Supplementary Material 2

## Data Availability

Data is provided within the supplementary information files”.

## References

[1] Berci G. In: MacFadyen BV, Arregui M, Eubanks S, Olsen DO, Peters JH, Soper NJ, et al. editors. Historical overview of surgical treatment of biliary stone disease. New York: Springer,Laparoscopic Surgery of the Abdomen; 2004. pp. 139–42.

[2] Gordon-Taylor. On gallstones and their sufferers. Br J Surg. 1937;25:241–51.

[3] Sun K, Soo KS, Seong C. Big data and analysis of risk factor for gallbladder disease in the young generation of Korea. PLoS ONE. 2019;14(2):1–13. 10.1371/journal.pone.0211480 PMC6386282 30794560

[4] Everhart JE. Burden of digestive diseases in the United States. Part III: liver, biliary tract and pancreas. Gastroenterology. 2009;136:1134–44. 19245868 10.1053/j.gastro.2009.02.038

[5] Angelico F, Barbato D-BM. Ten-year incidence and natural history of gallstone disease in a rural population of women in central Italy. GREPCO. Ital J Gastroenterol. 1997;29:249–54. 9646217

[6] TS. Epidemiology, pathogenesis and classification of biliary stones (common bile duct and intrahepatic). Best Pract Res Clin Gastroenterol. 2006;20:1075–83. 17127189 10.1016/j.bpg.2006.05.009

[7] Njeze GE. Gallstones. Nigerian J Surg. 2013;19(2). 10.4103/1117-6806.119236 PMC3899548 24497751

[8] Mohammad I, editor. Safe laparoscopic cholecystectomy an illustrated atlas. 1 ed. CRC; 2022. 2 Park Square,.

[9] Alelign T, Debella A, Tessema TS, Petros B. Thirteen years trend in the magnitude of urolithiasis and cholelithiasis in Ethiopia: evidence from hospital-based retrospective study. 2019.

[10] Adem A, Abdurahman AA. M, Pattern of surgical admissions to Tikur Anbessa Hospital, Addis Ababa, Ethiopia. East and Central Africa. J Surg 2001. 6(1).

[11] Raam Mannam RSN, Bansal A, Yanamaladoddi VR, Sai Suseel Sarvepalli, Shree Laya Vemula, Saikumar Aramadaka. Laparoscopic cholecystectomy versus open cholecystectomy in acute cholecystitis: a literature review. Cureus. 2023;15(9):e45704. 37868486 10.7759/cureus.45704 PMC10590170

[12] Blumgart LH, editor. BLUMGART’s surgery of the liver, biliary tract, and pancreas. 6th ed. Philadelphia: Elsevier; 2017.

[13] Rose JB. Diagnosis and management of biliary and injuries. Curr Probl Surg. 2017;54(8):406–35. 28987473 10.1067/j.cpsurg.2017.06.001

[14] Santos BFB. The difficult gallbladder: a safe approach to a dangerous problem. J Laparoendosc Adv Surg Tech. 2017;27(6):1. 10.1089/lap.2017.0038 28350258

[15] Veselin S, Nikola MM, Balsa K. A prospective cohort study for prediction of difficult laparoscopic cholecystectomy. Annals Med Surg. 2020;60:2. 10.1016/j.amsu.2020.11.082 PMC7779950 33425342

[16] HA. Difficult laparoscopic cholecystectomy: current evidence and strategies of management. Surg Laparosc Endosc Percutaneous Tech Am J Surg. 2011;21(4):211–7. 10.1097/SLE.0b013e318220f1b1 21857467

[17] Gupta N. Validation of a scoring system to predict difficult laparoscopic cholecystectomy. Int J Surg. 2013;11. 10.1016/j.ijsu.2013.05.037 23751733

[18] Ashish K, Khetan MY. Preoperative prediction of difficult laparoscopic cholecystectomy using a scoring system. Int Surg J, 2017;4(10)(3).

[19] Camilo R. Predicting the difficult laparoscopic cholecystectomy based on a preoperative scale. Updates Surg. 2022;74:3. 35122205 10.1007/s13304-021-01216-y PMC9213361

[20] Agrawal N. Preoperative prediction of difficult laparoscopic cholecystectomy: a scoring method. Nigerian J Surg. 2015;21(2). 10.4103/1117-6806.162567 PMC4566319 26425067

[21] Amin A. Preoperative and operative risk factors for conversion of laparoscopic cholecystectomy to open cholecystectomy in Pakistan. Cureus. 2019;11(8). 10.7759/cureus.5446 PMC6799874 31637145

[22] Hassan AM. Preoperative predictive risk factors of difficult laparoscopic cholecystectomy. Egypt J Surg. 2021;40:536–43.

[23] Lal P, Malik AP, Chakravarti VK. A difficult laparoscopic cholecystectomy that requires conversion to open procedure can be predicted by preoperative ultrasonography. JSLS. 2002;6:59–63. 12002299 PMC3043388

[24] Pujahari J. Preoperative prediction of diffi cult lap chole: a scoring method. Indian J Surg (July-August. 2009;71:198–201. 10.1007/s12262-009-0055-y PMC3452633 23133154

[25] Randhawa JS. Preoperative prediction of difficult lap cholea:scoring method. Ind J Surg. 2009;71:198–201. 23133154 10.1007/s12262-009-0055-y PMC3452633

[26] Rosen M. Predictive factors for conversion of laparoscopic cholecystectomy. Am J Surg. 2002;184(5). 10.1016/s0002-9610(02)00934-0 12354595

[27] Yasseina T. Predicting the risk factors of difficult laparoscopic cholecystectomy step by step. Egypt J Surg. 2020;39:515–22.

[28] Sugrue M. Grading operative findings at laparoscopic cholecystectomy- a new scoring system. World J Emerg Surg. 2015;10(14):4. 10.1186/s13017-015-0005-x PMC4394404 25870652

[29] Simopoulos C, Polychronidis BS, Tripsianis A, Karayiannakis G. Risk factors for conversion of laparoscopic cholecystectomy to open cholecystectomy. Surg Endosc. 2005;19:905–9. 15868267 10.1007/s00464-004-2197-0

[30] O’Leary D. Beware the contracted gall bladder: ultrasonic predictors of conversion. Surg. 2013;11(187):4. 10.1016/j.surge.2012.11.001 23287704

[31] Rattner DW, Warshaw FC. Factors associated withsuccessful laparoscopic cholecystectomy for acute cholecystitis. Ann Surg. 1993;217:233–6. 8452401 10.1097/00000658-199303000-00003 PMC1242774

[32] Rikki SINGAL, Muzzafar AS. The safety and efficacy of clipless versus conventional laparoscopic cholecystectomy – our experience in an Indian Rural Center. Mædica - a. J Clin Med. 2018;13(1):44–50. PMC5972786 29868139

[33] Muller MP, Marrie TG, Tang TJ, McGeer P, Low A. Can routine laboratory tests discriminate between severe acute respiratory syndrome and other causes of community-acquired pneumonia? Clin Infect Dis. 2005;40:1079–86. 15791504 10.1086/428577 PMC7107805

