# Supplementary material for: Validation of preoperative predictor score for difficult laparascopic cholecystectomy and a modified intraoperative grading score of the difficulty of laparascopic cholecystectomy: from a resource limited setting
Source: BMC Surg. 2025 Jan 27;25:42. doi: 10.1186/s12893-025-02784-1 (PMC11771005; doi:10.1186/s12893-025-02784-1)
Supplement: Supplementary file 2 — Supplementary Material 2 [file 12893_2025_2784_MOESM2_ESM.docx]

Questionnaire

Check list questionnaire for the analysis of preoperative and intraoperative predictors of difficult laparoscopic cholecystectomy at Yekatit 12 hospital medical college and St.PAULS millennium medical college , addis abeba, Ethiopia

The questionnaire developed with review of previous similar researches [[1-5](#_ENREF_1)]

**Part one** the following questions are the socio demographic characteristics and preoperative predictors of difficult laparoscopic cholecystectomy

|  | **Socio demographic characteristics and preoperative predictors of DLC** | | |
| --- | --- | --- | --- |
| **1** | Preoperative predictors | Sex | Male------------------------------1  Female --------------------------2 |
|  |  | Age (in yrs) | specify-------------------------- |
|  |  | BMI | <25  25-27.5  >27.5 |
|  |  | History of admission for acute cholecystitis | No  Yes |
|  |  | Palpable GB | YES  NO |
|  |  | Abdominal scar | YES (1.infraumblical 2. Supraumblical)  NO |
|  |  | Impacted stones | YES  NO |

Part Two: following questions are about intraoperative predictors of difficult laparoscopic cholecystectomy *Addis Ababa, Ethiopia*

|  | |  |
| --- | --- | --- |
| Intraoperative predictors | GB appearance | Adhesions < 50% of GB  Adhesions burying GB |
|  | Distended/contracted GB | Distended GB (or contracted shriveled GB)  Unable to grasp with atraumatic laparoscopic forceps  Stone ≥1 cm impacted in Hartman’s Pouch |
|  | Adhesions from previous surgery limiting access | No  Yes |
|  | Bile/puss outside of GB | No  Yes |
|  | Time to identify cystic artery/duct | <=90 minute  >90 minutes |
|  | BMI >30 | <=30  >30 |
|  | Adhesions from previous surgery limiting access | No: 0  Yes:1 |
|  |  |  |
|  | Bile / stone spillage | No  Yes |
|  | injury to duct or artery | No  Duct only  Both |
|  | Conversion to open | No  Yes |
|  | Ligature | Clip=0  Stitch=1 |

1. Ashish K. Khetan, M.Y., *Preoperative prediction of difficult laparoscopic cholecystectomy using a scoring system.* International Surgery Journal, 2017. **4(10)**(3).

2. Veselin S , M.M., Nikola K , Balsa S, *A prospective cohort study for prediction of difficult laparoscopic cholecystectomy.* Annals of Medicine and Surgery, 2020. **60**: p. 2.

3. Camilo R, *Predicting the difficult laparoscopic cholecystectomy based*

*on a preoperative scale.* Updates in Surgery, 2022. **74**: p. 3.

4. Gupta, N., *Validation of a scoring system to predict difficult laparoscopic cholecystectomy.* International Journal of Surgery, 2013. **11**.

5. Sugrue, M., *Grading operative findings at laparoscopic cholecystectomy- a new scoring system.* WORLD JOURNAL OF EMERGENCY SURGERY, 2015. **10(14)**: p. 4.
